# Studying the Expression Efficiencies of Human Clotting Factor IX Analogs, Rationally-designed for Hyper-glycosylation

**DOI:** 10.22037/ijpr.2020.112027.13503

**Published:** 2021

**Authors:** Fahimeh Ghasemi, M.Reza Khorramizadeh, Ali Asghar Karkhane, Alireza Zomorodipour

**Affiliations:** a *Department of Medical Biotechnology, Faculty of Medicine, Birjand University of Medical Sciences, Birjand, Iran.*; b *Department of Medical Biotechnology, School of Advanced Technology of Medicine, Tehran University of Medical Sciences, Tehran, Iran. *; c *Biosensor Research Center, Endocrinology and Metabolism Molecular-Cellular Sciences Institute, Endocrinology and Metabolism Research Institute (EMRI), and Zebrafish Core Facility-EMRI, Tehran University of Medical Sciences, Tehran, Iran. *; d *Institute of Industrial and Environmental Biotechnology (IIEB), National Institute of Genetic Engineering and Biotechnology (NIGEB), Tehran, Iran. *; e *Department of Molecular Medicine, Institute of Medical Biotechnology, National Institute of Genetic Engineering and Biotechnology (NIGEB), Tehran, Iran.*

**Keywords:** Human coagulation factor IX, Hyper-glycosylation, Mammalian expression system, CHO-s cell line, Site-directed mutagenesis

## Abstract

Glyco-engineering has attracted lots of interest in studies dealing with the pharmacokinetics of therapeutic proteins. Based on our previous *in-silico *studies, two sites were selected in the N-terminal gamma-carboxy glutamic acid-rich (Gla) domain of the human clotting factor IX (hFIX) to add new N-glycosylation sites. Site-directed mutagenesis was employed to conduct K22N and R37N substitutions and introduce new N-glycosylation sites in the mature hFIX. The expression efficiencies of the mutants, in parallel with the wild-type hFIX (hFIX^wt^), were assessed in suspension adapted Chinese hamster ovary (CHO-s) cells at transcriptional, translational, and post-translational levels. The transcription levels of both N-glycosylation mutants were significantly lower than that of the hFIX^wt^. In contrast, at the protein level, the two hFIX mutants showed higher expression. The occurrence of hyper-glycosylation was only confirmed in the case of the hFIX^R37N^ mutant, which decreased the clotting activity. The higher expression of the hFIX mutants at protein level was evidenced, which could be attributed to higher protein stability, via omitting certain protease cleavage sites. The coagulation activity decline in the hyper-glycosylated hFIX^R37N^ mutant is probably due to the interference of the new N-glycan with protein-protein interactions in the coagulation cascade.

## Introduction

Human clotting factor IX (hFIX) is a Vitamin K-Dependent (VKD) plasma glycoprotein, which takes part in the middle stage of the intrinsic pathway of blood coagulation (1, 2). It is synthesized by hepatocytes and secreted as a serine protease zymogen into the bloodstream. The circulating hFIX zymogen consists of 415 amino acids which are divided into four identifiable structural domains. The Gla domain (residues 1-45), with high affinity to Ca^2+^ ions, is located at the N-terminus of the mature hFIX. In the presence of the Ca^2+^ ions, the Gla domain undergoes structural changes, acquires stable conformation, and mediates the interaction of hFIX with phospholipids. The Gla domain is followed by two epidermal growth factor (EGF)-like domains (EGF1 and EGF2). A trypsin-like serine protease domain is located at the C-terminus of the hFIX, which is connected to the EGF2 via a 35-residue activation peptide (3-6).

Proteolytic cleavages at Arg^145^-Ala^146^ and Arg^180^-Val^181^ peptide bonds by either factor XIa (FXIa) or factor VIIa/tissue factor (FVIIa-TF) complex, in the presence of Ca^2+^ ions result in releasing of the activation peptide and subsequent formation of the physiologically active hFIX (hFIXa) (7). The hFIXa interacts with its co-factor, activated factor VIII (FVIIIa), in a Ca^2+^-dependent manner to form a tenase complex on the phospholipid membrane surface to catalyze the conversion of factor X (FX) to its active form (FXa) (8).

Deficiency or malfunctioning of hFIX leads to an X-linked congenital bleeding disorder, hemophilia B, with an incidence rate of one in 30000 live male births (5). Replacement therapy is the major treatment for the hemophilia B which is carried out via infusion of either plasma derived or recombinant hFIX (9). However, in recent years, concerns over the possible transmission of human blood-born infectious agents have motivated the use of recombinant forms of biotherapeutic products (10, 11). The structural complexity of multiple post-translational modifications (PTMs) such as VKD γ-carboxylation, β-hydroxylation, O-linked and N-linked glycosylation necessitates that the recombinant form of hFIX to be expressed in mammalian cells (12, 13). Different mammalian expression systems have been used to produce recombinant hFIX to ensure its appropriate PTMs, similar to those that occur in human cells (13, 14). Currently, the CHO cell line is preferably used for the production of therapeutic glycoproteins, including the hFIX. This cell line offers several advantages, in particular, production of glycoproteins with N-glycan similar to those found in human glycoproteins (15).

N-glycosylation is a co-or post-translational process that occurs on most of the secretory and membrane proteins in eukaryotic cells in the endoplasmic reticulum (ER) (16, 17), where the glucan moiety attaches to the Asn residue side chain located within the N-X-S/T sequon (where X can be any amino acid except proline) (17-19). N-linked glycans are an important part of glycoproteins and have a profound effect on protein properties such as folding, transmission, stability, solubility, degradation, and eventually protein secretion (16, 18 and 20). 

Glyco-engineering is a molecular approach to introduce new N-glycan(s) through the generation of new N-glycosylation site(s) within the amino acid sequences of the target proteins (21, 22). This process leads to hyper-glycosylation of the proteins and is mainly applied for the improvement of products of important biopharmaceuticals. Hyper-glycosylation may not only improve the pharmacokinetics of a therapeutic protein but in some cases, can increase the protein secretion by assisting its folding, especially when it occurs at C- or N-terminal region of a protein (18, 23 and 24). It has been demonstrated that secretion of a glycoprotein with an N-glycan close to the N-terminus can be improved by skipping the interaction with Bip and directly entering into the calnexin/calreticulin cycle (25). Secretion efficiency of a protein can be inversely correlated with the extent of Bip association in such a way that unglycosylated proteins display strong and stable associations with Bip (25, 26).

In this work, to investigate the impact of the hyper-glycosylation on the functional expression of the recombinant hFIX in CHO-s cells, the hFIX-encoding DNA was subjected for site-directed mutagenesis to introduce additional N-glycosylation sequon, somewhere in the N-terminal Gla domain, close to the protein N-terminus.

The candidate sites for hyper-glycosylation were selected based on the data obtained previously by molecular dynamic simulations of the 3D structure of the Ca^2+^ stabilized hFIX Gla domain (27). In this regard, two hFIX N-glycosylation mutants, namely; K22N and R37N were subjected for further expression analyses after being constructed via site-directed mutagenesis. Hyper-glycosylation occurrence, of the examined mutants and its impact on the functional expression of the hFIX was investigated at the various expression levels, including; transcription, translation, and post-translation.

## Experimental

*Materials and Methods*

*Site-directed mutagenesis and Construction of the recombinant expression plasmids*

**Figure 1:**
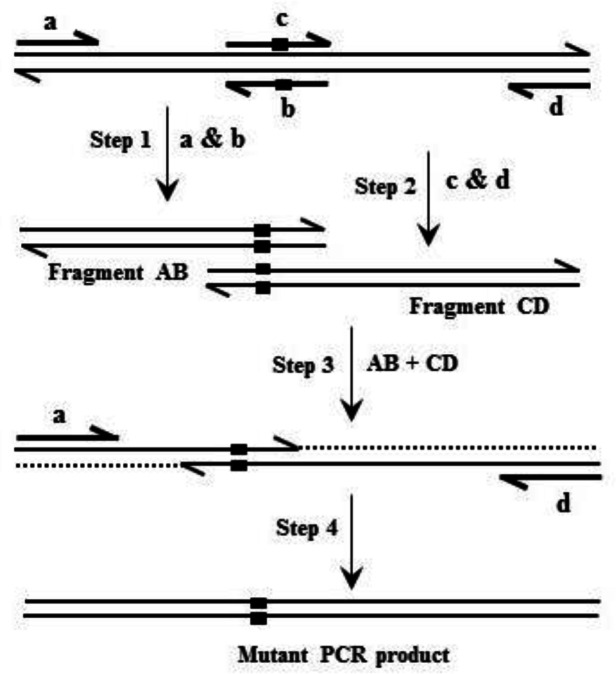
Schematic demonstration of the site-directed mutagenesis and construction of the hFIX N-glycosylation mutant expression plasmids

Based on overlap extension PCR (Figure 1), site-directed mutagenesis was applied to introduce mutations into the hFIX cDNA (28, 29). Oligonucleotides used for site-directed mutagenesis are listed in Table 1. Oligonucleotide pairs K22N-F/K22N-R and R37N-F/R37N-R were used respectively for introducing K22N and R37N substitutions into hFIX cDNA. Oligonucleotides hFIX-*EcoR*I and hFIX-*BamH*I equipped respectively with *EcoR*I and *BamH*I restriction sites, were used as forward and reverse primers for amplification of the pre-pro hFIX or its N-glycosylation mutants’ coding sequences. Primer hFIX-*BamH*I was equipped with a nucleotide sequence of 6xHis before the stop codon in order to introduce a His-tag to the recombinant proteins’ C-termini. The amplified fragments were then cloned into the pIRES2-EGFP vector, between *EcoR*I and *BamH*I restriction sites, upstream to the IRES-EGFP sequence.

Following *Nhe*I*/Not*I digestion, the target fragments, comprising the coding sequences of either the hFIX or its mutants and IRES-EGFP sequence, were subcloned into the pCEP4 expression vector (Thermo Fisher Scientific, USA), downstream to the cytomegalovirus (CMV) immediate early promoter/enhancer to end up with three bicistronic recombinant plasmids namely; pCEP4-hFIX^wt^, pCEP4-hFIX^K22N^, and pCEP4-hFIX^R37N^.

*Cell Culture, Transfection, and Transient Expression*

Suspension adapted Chinese hamster ovary (CHO-s) cell line (Thermo Fisher Scientific, USA), considered as a mammalian expression host, was cultured in FreeStyle™ CHO Expression Medium (Thermo Fisher Scientific, USA) supplemented with 8mM L-glutamine (Thermo Fisher Scientific, USA) and incubated at 37^o^C in an 8% CO_2_ atmosphere while shaking at 140 rpm. For transient expression, the cells were transfected with bicistronic recombinant plasmids; pCEP4-hFIX^wt^, pCEP4^K22N^, and pCEP4^R37N^ (expressing either wild-type or mutant forms of the hFIX, together with an enhanced green fluorescent protein, EGFP, as a reporter protein), using FreeStyle™ MAX reagent (Thermo Fisher Scientific, USA), according to the manufacturer’s instruction. Three to five h after transfection, vitamin K (Caspian Tamin, Iran) was added at a final concentration of 2.5µg/mL. At 24, 48, and 72 h post-transfection, the cells were pelleted and the culture media were harvested and subjected for expression analyses**.**

*Transcript Stability analysis*

Secondary structures of the mRNAs from complete sequences of the hFIX^wt^ and its N-glycosylation mutants’, were predicted using Mfold Web Server (http://unafold.rna.albany.edu/?q=mfold) (30). The analyzed regions included the coding sequences of the hFIX^wt^ or its mutants and the IRES-EGFP sequence.

*RNA Extraction and cDNA Synthesis*

Total RNAs were isolated from the transfected CHO-s cells at 24, 48, and 72 h post-transfection using high pure RNA isolation Kit (Roche, Germany), according to the instruction provided by the manufacturer. Both quantity and purity of the total isolated RNAs were determined by UV absorbance measurement at 230, 260, and 280 nm by NanoDrop spectrophotometer. OD_260_/OD_280_ ratio of ~2 and OD_260_/OD_230_ ratio of 2-2.2 were considered as an acceptable purity for the RNA samples.

The integrity and quality of the purified RNAs were further checked using 2% agarose/formaldehyde electrophoresis and ethidium bromide staining. Then 1 µg of total RNA was treated with DNase I to eliminate genomic DNA and plasmids contaminations, and subjected for the first strand cDNA synthesis using oligo dT primers and revertAid M-MulV reverse transcriptase (Thermo scientific, USA). 

*Real-Time Quantitative PCR (qPCR)*

The synthesized cDNAs were then used as a template for the subsequent qPCR experiment using hFIX-specific oligonucleotide hf9-RTF1 and hf9-RTR1 as forward and reverse primers, respectively (Table 1). A 117 bp fragment from hamster glyceraldehyde 3-phosphate dehydrogenase (Gapdh; a housekeeping gene) gene was amplified as an internal control using hams-gapdhF1 and hams-gapdhR1 primers (Table 1). To quantify the relative mRNA level of each construct, SYBR green-based real-time PCR was carried out using RealQ Plus Master Mix Green (Ampliqon, Denmark) on Corbett Rotor-Gene 6000 Real-Time PCR machine (Qiagen, USA). The recombinant gene transcription was normalized to that of the Gapdh gene. About 50 ng cDNA sample, 0.5 μL of each forward and reverse primer (10 µM) and 5 μL RealQ Plus Master Mix Green (Ampliqon), in a final volume of 10 μL, were used for real-time PCR. The qPCR amplification temperature profile was as follow: 95 °C for 5 min (denaturation), followed by 40 cycles of amplification for 20 s at 95 °C, 20 s at 61 °C, and 20 s at 72 °C. All qPCRs were performed in triplicates. A reaction sample without cDNA was performed as a control in each run. Gapdh housekeeping gene was used as an internal control for normalization. Specificity of the primer pairs and the purity of real-time PCR products were confirmed by melting curve analysis of 65 °C to 95 °C, with fluorescence measured every 0.5°C at the end of each reaction (with no nonspecific and primer-dimer formation). Gel electrophoresis was performed to approve the PCR products and primer specificity.

*Flow Cytometry Analysis*

CHO-s cells transfected with bicistronic recombinant plasmids, expressing hFIX^wt^ or its N-glycosylation mutants along with EGFP as a reporter protein, were observed using fluorescence microscope 24, 48, and 72 h after transfection to detect EGFP signals. The transfection efficiencies were then monitored using flow cytometric analysis. About 2 × 10^5^ CHO-s cells were harvested by centrifugation at 100 ×g for 5 min, and washed twice with PBS. The cytosolic expression of EGFP in transfected cells were analyzed using flow cytometry (BD FACSCalibur, USA). EGFP fluorescence was excited at 488 nm, and emission was measured with a 530/30 nm bandpass filter. Untransfected cells were served as a control for autofluorescence and were used to set the gate for positive cells to exclude debris. The results were analyzed using FlowJo software (Treestar, Inc.).

*Quantification of the Recombinant hFIX Accumulated Inside the CHO-S Cells*

After 72 h of transfection, 5 × 10^6^ CHO-s cells were pelleted by centrifugation at 100 g for 5 min. The pellets were then re-suspended in 500 μL of ice-cold lysis buffer (50 mM Tris, pH 8, 150 mM NaCl, 0.1% triton X-100, 0.5% sodium deoxycholate, 0.1% SDS, containing protease inhibitor mix (Roche). After 20-30 min, the lysate was centrifuged at 12000 ×g for 20 min at 4 ºC, and the supernatant was analyzed for intra-cellular hFIX antigen, using ELISA. 

*Enzyme-Linked Immunosorbent Assay (ELISA) of hFIX Antigen (hFIX::Ag) in Culture Media*

Culture media were subjected for quantification of hFIX^wt ^or its N-glycosylation mutants using standard ELISA, based on the procedure provided by the manufacturer (Asserachrom, France). To quantify intracellular expression of recombinant hFIX^wt^ or mutants, 50 µL of cell lysate was used for ELISA assay. The concentration of the expressed hFIX^wt^ and mutants in culture media or intra-cell were calculated based on the standard curve and stated in ng/10^6^ transfected cells. 

*Coagulation Assay*

The coagulation activity of the secreted hFIX^wt^ and its N-glycosylation mutants were measured using a chromogenic assay, according to manufacturer’s protocol (Biophen factor IX kit, France). The citrated human plasma derived from normal individuals was used as a standard sample. The culture medium from untransfected cells was used as a negative control.

*The hFIX Purification*

Recombinant proteins expressed by CHO-s cells transfected with bicistronic expression plasmids were then purified using Ni-NTA purification system. After 72 h of transfection, the cell suspensions were centrifuged at 100 g for 5 min and culture media were harvested. About 15 mL of the culture media was subjected for affinity purification using Ni-NTA beads (provided by Dr. Mohammadi at NIGEB, Iran).

*SDS-PAGE and Western Blotting*

The purified recombinant hFIX and mutants were analyzed using 4–12% gradient SDS-PAGE (31) to characterize hyper-glycosylated mutants in comparison with the native hFIX. Electroblotting of proteins onto polyvinylidene difluoride (PVDF) membrane (Roche, Germany) was performed using a wet procedure, in a transfer buffer (25 mM Tris, 192 mM glycine, 20% methanol) for 3 h at 300 mA. 

The blot was then blocked in 5% non-fat milk solution (5% w/v, in Tris-buffered Saline containing 0.1% Tween 20) and subjected for Western blotting experiment during which the electroblotted protein bands were probed with a rabbit polyclonal anti-hFIX primary antibody (Abcan, UK) with dilution of 1/2500 which was followed by incubation with goat anti-rabbit secondary antibody conjugated with peroxidase (Millipore, Co. US) with a dilution of 1/2500. Immunoreactive bands were visualized by enhanced chemiluminescence (ECL) Western blotting detection kit (Najm, Iran) or by 4-chloronaphtol substrate. 

*Enzymatic Deglycosylation*

The purified wild-type and mutant rhFIX samples were subjected for deglycosylation using PNGase-F enzyme kit (Biolabs, New England) according to the manufacturer’s protocol. Following the denaturation step, the protein samples were treated with PNGase-F enzyme in reaction buffer containing 1% NP-40. Deglycosylated samples were then examined by 4-12% gradient SDS-PAGE and Western blotting analysis.

*Statistical Analysis*

All experiments were done in triplicates. The values were expressed as means±SD. The statistical analyses were performed by one-way analysis of variance (ANOVA) followed by Duncan post hoc using SPSS version 22 software. The P values less than 0.05 were considered to be statistically significant.

## Results

*Transfection Efficiency Analysis*

**Figure 2:**
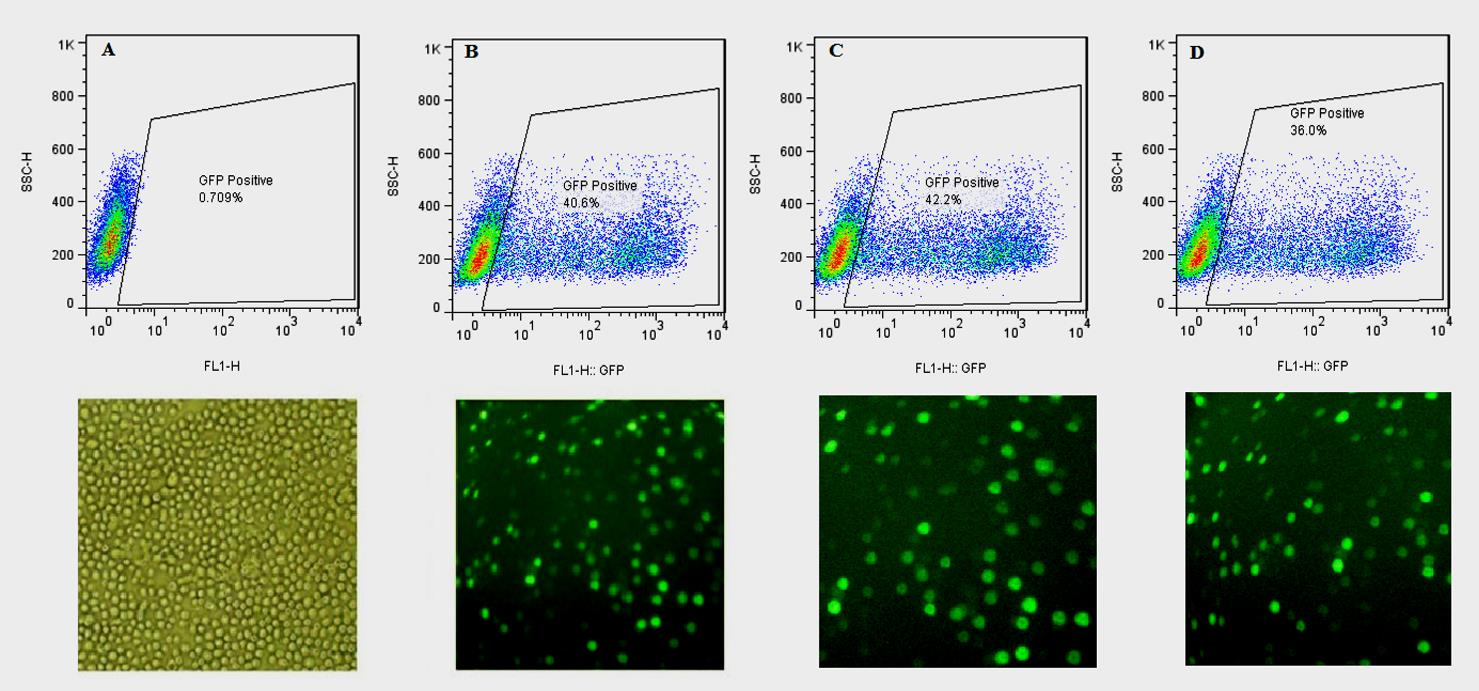
Photograph of the transfected CHO-s cells obtained with a fluorescence microscope,at magnification of 10X, and the rate of transfection obtained with flow cytometry,24 h after transfection. (A) Untransfected CHO-s cells, (B) CHO-s cells transfected with pCEP4-hFIX^wt^, (C) CHO-s cells transfected with pCEP4-hFIX^K22N^, (D) CHO-s cells transfected with pCEP4-hFIX^R37N^ plasmids

The three bicistronic plasmids, expressing either the wild-type or one of the two N-glycosylation mutants (hFIX^K22N^ and hFIX^R37N^(, along with EGEF, were separately applied for transient transfection of CHO-s cells. The transfection efficiency was evaluated using flow cytometry at 24, 48, and 72 h post-transfection. The untransfected cells were used as a control to exclude autofluorescence (Figure 2). All the examined hFIX mutants contained the hFIX native signal peptide; therefore, they were expected to be secreted into the culture media, whereas the expressed EGFP remained in the cytosol, and the cells appeared in green color under fluorescence microscopy (Figure 2). 

*The hFIX Transcript Analysis*

Transcription of the recombinant hFIX^wt ^and its mutants in transiently transfected CHO-s cells were confirmed by performing RT-PCR and qPCR, at different post-transfection time points (24, 48, and 72 h). Electrophoresis pattern of the amplified cDNA by hFIX specific primers yielded a PCR product of 117 bp. Hamster’s Gapdh gene was also amplified using internal specific primers to produce 116 bp PCR products. 

**Figure 3.**
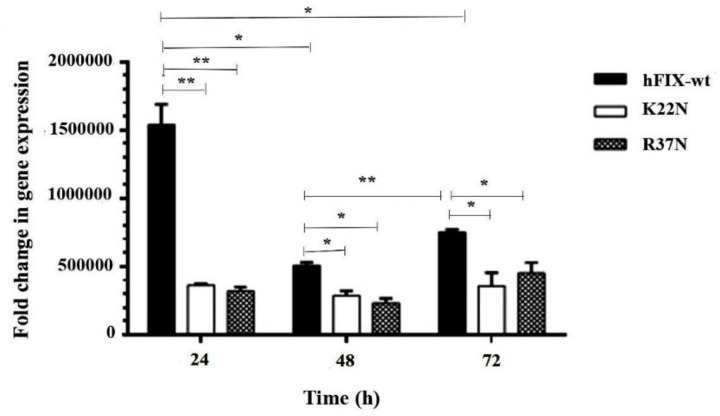
qPCR analysis of transcripts from hFIX^wt^ and two N-glycosylation mutants 24, 48 and 72 h after transfection. Asterisks indicate samples that are signiﬁcantly different (*p* < 0.05) compared to other samples, using analysis of variance. ^*^p ≤ 0.05, ^**^p ≤ 0*.*01

In the continuation of the transcripts’ analysis, comparative qPCRs were performed on the mutants to investigate the variation of hFIX mRNA levels due to the mutations (Figure 3). Normalization was carried out using amplification of hamster’s Gapdh gene. As the results revealed, the transcription level for hFIX^wt^ was significantly higher than those of the examined mutants at 24 h post-transfection. In fact, the highest transcription expression level occurred for the wild-type construct 24 h after transfection. No significant differences between the mutants at transcription expression levels were observed (Figure 3).

*Immunoassay of the Expressed hFIX Mutants*

**Figure 4:**
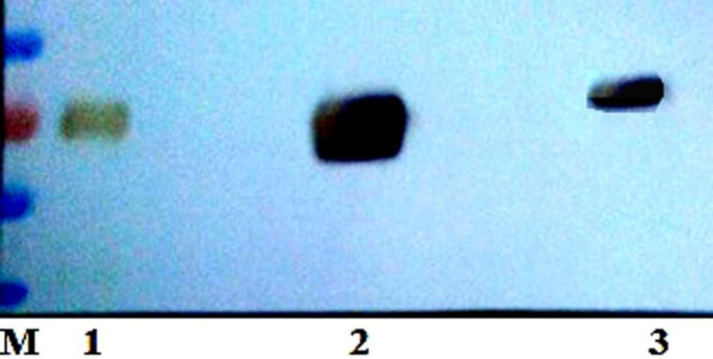
Western blotting analysis of the purified wild-type and mutant forms of hFIX expressed by transiently transfected CHO-s cells. Lane 1, protein molecular weight, Lane 2: hFIX^wt^, Lane 3, hFIX^K22N^ mutant, Lane 5, and hFIX^R37N^ mutant

Considering that N-glycosylation often drastically increases the molecular weight of a protein, the two hFIX N-glycosylation mutants and the hFIX^wt^ were subjected for gradient SDS-PAGE and Western blotting analysis after purification using Ni-NTA beads. The three samples were detectable with the specific anti-hFIX antibody. The hFIX^R37N^ showed a higher molecular weight than the hFIX^wt^ and hFIX^K22N^ (Figure 4), suggesting the probability of the hyper-glycosylation occurrence in the case of the hFIX^R37N^. 

**Figure 5:**
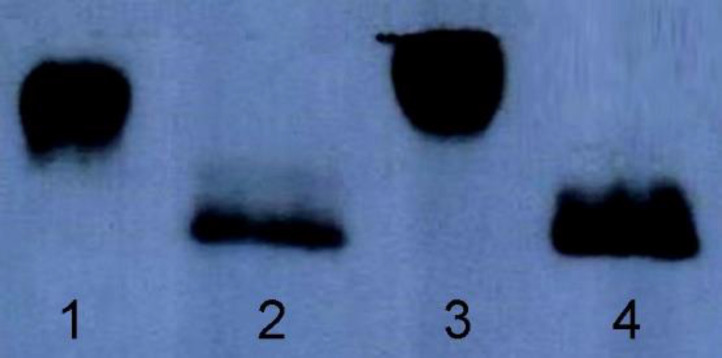
Western blotting analysis of the hFIX^wt^ and the hFIX^R37N^ before and after PNGase digestion. Lane 1: hFIX^wt^ before digestion with PNGase, lane 2: hFIX^wt^ after digestion with PNGase, lane 3: hFIX^R37N^ mutant before digestion with PNGas, lane4: hFIX^R37N^ mutant after digestion with PNGase

In order to confirm the correlation between the increased molecular weight of the hFIX^R37N^ and N-glycan attachment, the hFIX^R37N^ along with the hFIX^wt^ were treated with PNGase-F and examined using gradient SDS-PAGE and Western blotting analysis (Figure 5). PNGase-F is an amidase that catalyzes the cleavage between the innermost GlcNAc and asparagine residues from *N*-linked glycoproteins (32). As shown in the Western blotting results, both samples migrated at the same molecular weight after being treated with PNGase-F (Figure 5). Indeed, an expected gel-shift upon similar treatment of both wild-type hFIX and hFIX^R37N^ mutant with PNGase-F was observed and the same protein size on the blot was obtained (Figure 5, lanes 2 and 4).

**Figure 6.**
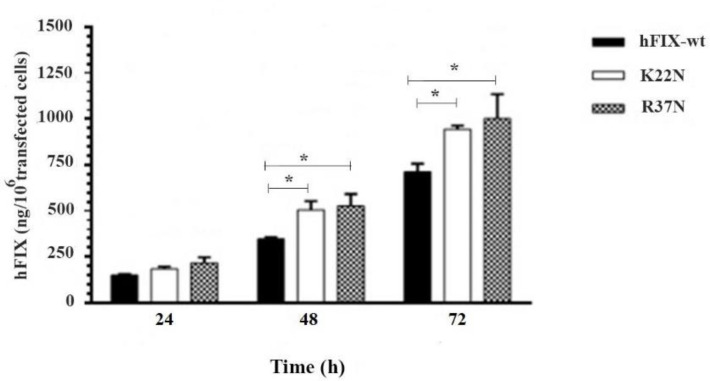
Evaluation of transient expression of hFIX^wt^ and its N-glycosylation mutants, hFIX^K22N ^and hFIX^R37N, ^in CHO-s cells at various post-transfection time (h), based on ELISA performed on the samples taken from culture media. Asterisks indicate samples that are signiﬁcantly different (*p *< 0.05) compared to other samples, using analysis of variance

^*^p ≤ 0.05, ^**^p ≤ 0.01.

*Assessment of the hFIX Expression Level Based on the ELISA *In order to examine the protein expression levels of the wild-type hFIX and its mutants, during 72 h after transfection, the culture media were harvested at three time points, 24, 48, and 72 h post-transfection, and subjected for hFIX expression analysis using ELISA. Based on the results obtained, the wild-type hFIX and mutants were all recognized by the hFIX antibody. At 48 and 72 h post-transfection, both mutants showed a significant increase in the hFIX expression level, when compared with that of the hFIX^wt^ (Figure 6). However, the highest hFIX expression levels for all three constructs occurred on the 3^rd ^day after transfection. 

*Evaluation of Secretion Efficiency of the Expressed hFIX* ^wt^ *and mutants*

**Figure 7:**
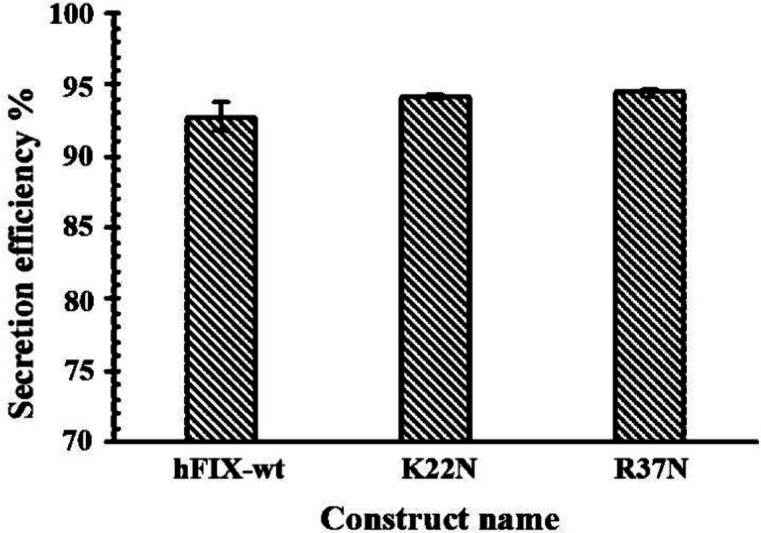
The hFIX Secretion efficiency by CHO-s cells 72 h post-transfection by hFIX^wt^, hFIX^K22N ^and hFIX^R37N^ constructs

Secretion efficiency of a protein is the ratio between the secreted fraction of the protein and its total amount (secreted plus intracellular fractions) (33). In order to calculate the hFIX secretion efficiencies for the examined constructs, their corresponding cell lysates and culture media were harvested 72 h after transfection and considered for secretion efficiency assessment. Analysis of the hFIX level in cell lysates and calculation of secretion efficiencies of the examined constructs indicated no significant differences between secretion efficiencies of the mutants and their wide-type counterpart (Figure 7). 

*Measurement of Coagulation Activity of the Secreted hFIX*

**Figure 8.**
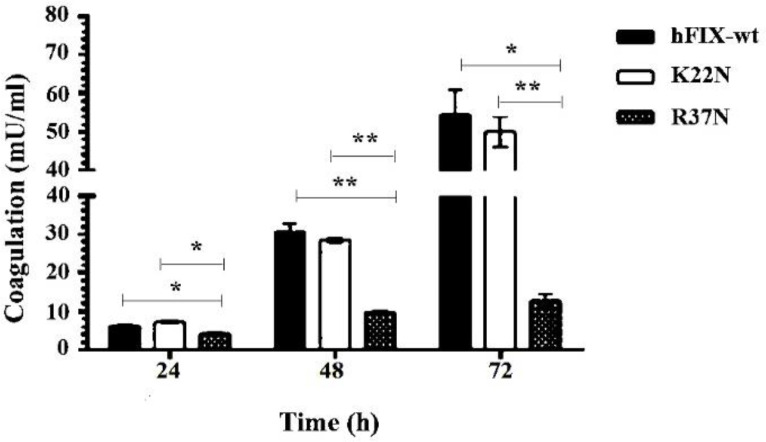
Coagulation assay for hFIX^wt^ in comparison with the N-glycosylation mutants; hFIX^K22N^ and hFIX^R37N^, during 72 h after transfection. Asterisks indicate samples that are signiﬁcantly different (*p *< 0.05) compared to other samples, using analysis of variance. ^*^p ≤ 0.05, ^**^p ≤ 0.01

The coagulation activities of the hFIX^wt^ and N-glycosylation mutants in the culture media were measured by chromogenic assay at different time points. Our results showed a reduced clotting activity for the hFIX^R37N^ mutant, compared to that of the hFIX^wt^ (Figure 8). No significant difference was observed between clotting activities of the hFIX^K22N^ mutant and the hFIX^wt^ (Figure 8). 

*The Prediction of the Transcript Stabilities*

The mRNA stabilities for the wild-type and mutants in the form of bicistronic mRNAs were obtained through calculating free ΔG. The free ΔGs for three mRNA were -913.20, -915.70, and -915.40 for hFIX^wt^, hFIX^K22N^, and hFIX^R37N^ respectively, which showed no significant differences among them. 

## Discussion

Based on the previously described rational methodologies (27), two positions were selected to introduce new N-glycosylation sites in the Gla domain of the hFIX by means of site-directed mutagenesis and single amino acid substitution. Introducing a new N-glycosylation site does not ensure the glycan attachment to Asn residue located in there (18). In order to investigate the N-glycosylation occurrence in the newly introduced N-glycosylation sites of two hFIX mutants, the culture media from the transfected cells were collected and subjected for gradient SDS-PAGE. The hFIX^R37N^ mutant showed higher molecular weight that the hFIX^wt^, implying that hyper-glycosylation might occur, while the hFIX^K22N^ mutant demonstrated the same molecular weight as the wild-type. After PNGase treatment of the hFIX^R37N^ in parallel with the wild-type, the hFIX^R37N ^mutant acquired the same size as that of the hFIX^wt^. This finding confirmed the creation of a new N-glycosylation site in the hFIX^R37N^ mutant, recognizable by oligosaccharyl transferase for an N-glycan attachment to Asn^37^ residue.

Failure of hyper-glycosylation in the case of the hFIX^K22N^ mutant, can be explained by the participation of the Cys^23^, located at +1 position in the newly generated N-glycosylation site (*i.e. *Asn^22^-Cys^23^-Ser^24^), in a disulfide bond formation with the Cys^18^, generating a loop (34, 35). The generated loop, which entraps the N-glycan acceptor Asn^22^, might prevent the attachment of the oligosaccharide to Asn^22^ residue. Previously it has been reported that N-glycosylation and disulfide bond formation have reciprocal effects so that a glycan attachment may sterically hinder the formation of a disulfide bond, and a disulfide bond formation, in turn, may prevent the oligosaccharide attachment to the nearby N-glycosylation site (36). In an experiment carried out by McGinnes and coworkers on hemagglutinin-neuraminidase (HN), a glycoprotein of the Newcastle disease virus (paramyxovirus), either of the cysteine residues flanking an unused N-glycosylation site was removed by substitutional mutation, leading to the attachment of a glycan (37). In this experiment, site-directed mutagenesis was carried out to remove either of the two cysteine residues participating in the formation of a disulfide bridge near the unused N-glycosylation site. Their results indicated the attachment of oligosaccharide after the removal of the disulfide bond. They concluded that the disulfide bond formation nearby the N-glycosylation site might prevent the glycan attachment (37). 

In the case of hFIX^K22N^ mutant, a serine residue at position +2 in the Asn^22^-Cys^23^-Ser^24 ^N-glycosylation-site, which is reported to be less effective than a threonine in the attachment of glycan to the Asn residue, might prevent the N-glycosylation (22). 

Comparison of the expression efficiencies of the two hFIX mutants with that of the wild-type, at transcriptional and translational levels, led to different outcomes. At transcription level, the highest expression was related to the wild-type hFIXat 24 h after transfection, while at protein levels both mutants had higher expression than the wild-type at 48 and 72 h post-transfection. Since there were no significant differences among the ΔGs of the transcripts, the higher protein expression observed for both mutants cannot be attributed to the stability of their transcripts. 

Our previous *in-silico* studies indicated in the increased stabilities for both mutants, hFIX^K22N^ and hFIX^R37N ^(27). In agreement with the *in-silico* studies, the higher protein expression levels were demonstrated for the two hFIX mutants in comparison with the hFIX^wt^, which might be due to their higher proteolytic stabilities, and their accumulations during the time courses of the protein production. In fact, the higher stabilities of the hFIX mutants in this work can be due to substitutions of Asn for Lys or Arg, in the hFIX^K22N^ and hFIX^R37N^, respectively, and elimination of cleavage sites for a number of proteases including; LysC, LysN, trypsin, and Arg-C (38). 

Comparison of the coagulation activity of the hFIX^K22N^ mutant with that of the hFIX^wt ^showed no significant difference. However, the hyper-glycosylated mutant, hFIX^R37N^, showed a reduced clotting activity when compared with that of the hFIX^wt^. This result indicated that the attachment of new N-glycan interferes with the enzymatic activity of the hFIX. The new N-glycosylation site in this hyper-glycosylated mutant is located within the protein Gla domain, which participates in the interaction of the protein with coagulation factor XI, another important protein in coagulation cascade, as well as phospholipid bilayer (39). It was shown that hyper-glycosylation of proteins in some cases increase the protein secretion by assisting its folding (18, 23 and 24). However, this was not the case for the hFIX mutants examined in this work. The effect of N-glycans close to the N- or C-terminal ends of a protein on its secretion efficiency is protein dependent and varies among different protein (18). Although relatively higher expression levels were observed for both of the hFIX mutants, regardless of their opposite outcomes in receiving an extra N-glycan, their secretion efficiencies were not affected by the mutation in CHO-s cells. 

**Table 1:** The primes used for qPCR, mutagenesis and amplification of the coding sequences of the hFIX^wt^ and its N-glycosylation mutants, the restriction sites are underlined

**primers**	**Nucleotide sequences (5´ → 3´)**	**Restriction site**
hFIX-*EcoR*I	CCGGAATTC GCC ACC ATG CAG CGC GTG	*Eco*RI
hFIX-*BamH*I	CGCGGATCCTCATTAAGTGAGCTTTGTTTT	*Bam*HI
K22N-F	TGTATGGAAGAAAACTGTAGTTTTGAAGAAGC	-
K22N-R	GCTTCTTCAAAACTACAGTTTTCTTCCATAC	-
R37N-F	GAAAACACTGAAAACACAACTGAATTTTGG	-
R37N-R	CCAAAATTCAGTTGTGTTTTCAGTGTTTTC	-
hf9-RTF1		
hf9-RTR1		
hams-gapdhF1		
hams-gapdhR1		

## Conclusion

In accordance with our previous *in-silico* prediction, hyper-glycosylation of hFIX in the new N-glycosylation site, introduced by R37N amino acid substitution, was approved. Regardless of the N-glycan attachment in the case of both mutants, their expression in protein level was increased in comparison with the hFIX^wt^. No significant difference was observed between the free ΔGs of the transcripts, and the secretion efficiencies of the hFIX mutants in CHO-s cells were not affected by the mutations. Therefore, the improvement of the expression efficiencies of the hFIX mutants, at protein level could be attributed to enhanced protein stability, via omitting certain protease cleavage site. The hFIX^R37N^ mutant showed a decreased clotting activity when compared with that of the hFIX^wt^, probably due to interference of the new N-glycan with the enzymatic activity of the hFIX^R37N^. 
